# Management of symptomatic patients with suspected mild-moderate COVID-19 in general practice. What was published within the first year of the pandemic? A scoping review

**DOI:** 10.1080/13814788.2021.2002295

**Published:** 2021-11-18

**Authors:** Anne Holm, Anne Møller, Rune Aabenhus

**Affiliations:** Research Unit for General Practice, Department of General Practice, University of Copenhagen, Copenhagen, Denmark

**Keywords:** General practice, COVID-19, infections, primary care

## Abstract

**Background:**

Most COVID-19 patients experience a mild course of the disease and can be managed in general practice. However, in the early pandemic, most research was conducted in secondary care.

**Objectives:**

This scoping review aimed to identify original research published within the first year of the pandemic relevant to general practice regarding symptomatic, non-hospitalised patients with mild to moderate COVID-19 disease to provide an overview of published research.

**Methods:**

PubMed was searched for studies written in English, Swedish, Danish, or Norwegian published before 1 April 2021. Two authors screened all titles and abstracts and identified full texts.

**Results:**

We screened 1303 titles and abstracts and retrieved 128 full texts. An additional 44 full-texts were obtained from references. After full-text reading, 79 articles were included, six of which were conducted in general practice, 20 in the community, 42 in hospitals, and 11 in other settings. Therapy and harm were investigated in randomised controlled trials in 11 out of 17 studies; the diagnosis was investigated using a diagnostic accuracy design in four out of 26 studies and prognosis in prospective studies in 10 out of 21 studies. The remaining 15 studies had other research questions.

**Conclusion:**

Although general practitioners in most countries must have been involved in managing patients with COVID-19, little research has been published from general practice during the first year of the pandemic. General practice research environments must be able to respond quickly in case of future pandemics.

KEY MESSAGES

- Several studies have been published regarding patients with mild-moderate COVID-19 within the first year of the COVID-19 pandemic but few of these have been conducted in general practice.
- This finding should give rise to reflection on how to handle research on future pandemics in general practice research environments.

## Introduction

Since the coronavirus disease 2019 (COVID-19) was declared a pandemic by WHO on 11 March 2020, the number of research articles regarding severe acute respiratory syndrome coronavirus 2 (SARS-CoV-2) and COVID-19 have surged [1,2].

The pandemic caused a steep increase in admissions of severely and critically ill patients, at times leaving overburdened hospitals in some parts of the world temporarily unable to attend to all COVID-19 patients. To minimise the strain on secondary and tertiary care facilities, effective management in general practice is essential to correctly triage and distinguish between acutely ill patients needing hospital admission and mild to moderate cases. Especially since 80% of COVID-19 cases are considered mild to moderate in intensity and generally do not require hospitalisation [3,4]. In Denmark, as well as many other countries, general practitioners (GPs) are the first point of contact when sick patients need advice or assessment in the health care system. However, in the initial phases of the pandemic, the main focus of most clinicians and researchers was on critical care management, and the majority of early research studies included only hospitalised patients or patients with severe COVID-19 [5,6]. To correctly triage and advise patients, it is essential that high-quality evidence relevant to general practice patients is available. However, it is difficult to obtain an overview of which relevant patient populations and research questions have already been investigated and if this research has been conducted in general practice or outpatient departments due to the restructuring of health systems during the pandemic. Despite the rollout of effective vaccines, the COVID-19 continues to return in pandemic waves due to new SARS-CO-2 variants and lack of immunity in the populations, causing adaptations in clinical practice to counter the surges.

This scoping review aimed to identify original research published within the first year of the pandemic relevant to general practice regarding symptomatic, non-hospitalised patients with mild to moderate suspected or confirmed COVID-19 disease to provide an overview of published research and identify gaps of knowledge.

## Methods

### Literature search

We searched PubMed for studies published before 1 April 2021. The suggested search string published on the front page of PubMed during the first six months of the pandemic to identify studies on COVID-19 was used in combination with the words ‘mild’ and ‘moderate’ as well as a slightly modified version of a previously validated search string to identify studies from general practice or primary care [7]. The full search string can be seen in Supplementary Appendix 1. The search was conducted in two rounds. An original search in December 2020 and an updated search after submitting the first article draft in May 2021.

### Selection of eligible studies

Two authors (AH and either RA or AM) screened titles and abstracts and selected full texts for eligible studies.

#### Patient population

We accepted studies on symptomatic non-hospitalised patients with suspected or confirmed mild to moderate COVID-19.

#### Design

We accepted clinical studies regardless of study design. Quantitative studies had to include at least 50 patients with suspected or confirmed COVID-19.

#### Setting

We sought to include studies from primary care, but since patients suspected of COVID-19 have been assessed and treated in various locations during the pandemic, we accepted any study on patients that were not hospitalised at the time of clinical evaluation. This included studies from outpatient clinics, quarantine stations, testing sites, and the community. Studies were excluded if they involved tests not widely available in general practice (e.g. CT-scans). We accepted studies including both hospitalised patients and outpatients if the two groups were reported separately and the outpatient group included at least 50 patients.

#### Language and publication

We accepted articles written in English, Danish, Swedish, or Norwegian. Preprints were excluded.

#### Relevance

We excluded studies fulfilling all of the above criteria if they had no findings to report of relevance to GPs.

Reference lists of included studies were screened for possible additional new studies to include. In case of disagreement, the third author (AM or RA) was consulted. We did not exclude studies due to poor quality.

### Data extraction

We extracted the following data from each included article: Date (e)published, Title, Country, Study setting, Patients population(s), fulfilling inclusion criteria, Research question, Topic, Design, and Main findings Data extraction was performed by two authors (AH and either RA or AM). In case of disagreement, the third author (RA or AM) was consulted.

### Synthesis of studies

We grouped the main purpose of the study endpoints into five predefined topics: ‘therapy’, ‘harm’, ‘diagnosis’, ‘prognosis’, and ‘other’. Similarly, we predefined the following six study designs: ‘randomised controlled trial (RCT)’, ‘diagnostic accuracy’, ‘observational–prospective’, ‘observational–retrospective’, ‘mixed-methods’, and ‘qualitative’. New topics and study designs could be added during data extraction if needed. Grouping of categories and study designs were done according to the principles of evidence-based medicine [8]. Settings and patient populations were categorised while extracting data.

### Synthesis of main findings and quality assessment

We planned a non-systematic, narrative summary of the main findings of the included studies. We did not plan a quality assessment besides summarising which study designs were used for which research questions.

## Results

**Figure 1.**
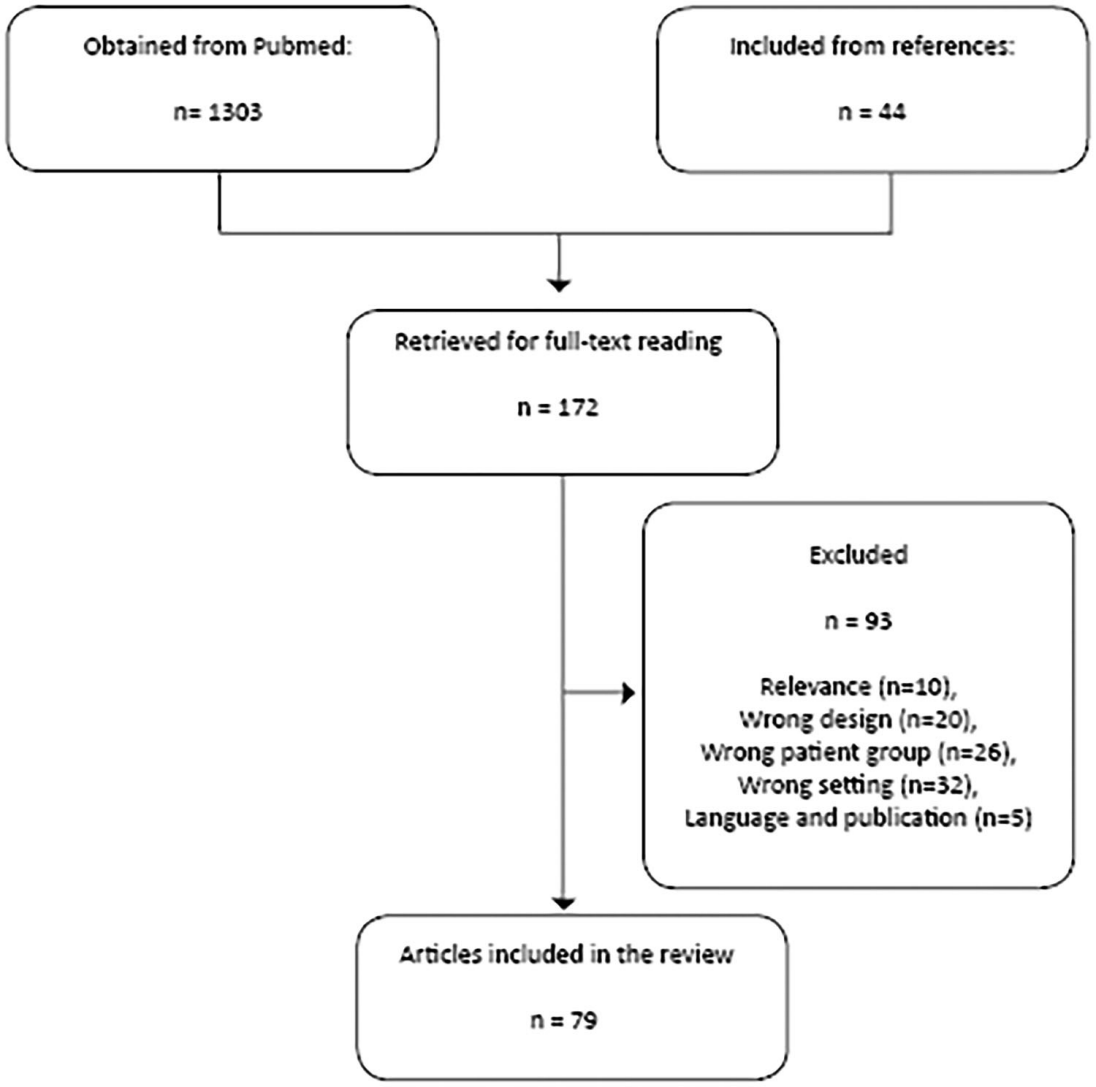
Inclusion flow chart.

The literature search resulted in 1303 unique entries published before 1 April 2021 (May 2021 search). After the screening of titles and abstracts, 128 unique articles were retrieved for full-text reading. Screening of references of included articles resulted in additional 44 articles. After full-text reading, 79 articles were included in this review. The reasons for exclusion were Relevance (*N* = 10), Wrong design (*N* = 20), Wrong patient group (*N* = 26), Wrong setting (*N* = 32), and Language and publication (*N* = 5) (Figure 1).

Table 1 shows the settings and patient populations of the included studies. Twenty studies were conducted in the community, six in general practice, 42 in hospital outpatient settings or on hospital staff, seven in quarantine stations or at testing sites, and four in other primary care settings. Most studies investigated either non-pregnant adults (*N* = 59) or healthcare workers (*N* = 12).

**Table 1.** Settings and patient populations of the included studies.

	Number of studies	Median number of patients (range when applicable)
Community	20	297.5 (13–4182)
Adult patients	9	400 (87–4182)
Adult patients with prior infection	8	548 (24–2112)
Adults and children	1	180
Healthcare workers	1	293
Healthcare workers with prior infection	1	13
General practice	6	353.5 (61–743)
Adult patients	3	73 (61–518)
Adult patients with prior infection	1	743
Adults and children	1	360
Adults and children with prior infection	1	347
Hospital (outpatient or staff)	42	220 (50–12,022)
Adult patients	28	205 (50–2733)
Children	1	94
Healthcare workers	9	803 (132–12,022)
Healthcare workers with prior infection	1	160
Pregnant patients	3	158 (132–232)
Other primary care settings	4	129.5 (57–237)
Adult patients	3	167 (57–237)
Pregnant patients	1	92
Quarantine station or testing site	7	277 (60–3890)
Adult patients	7	277
Total	79	237 (13–12,022)

All of the five predefined topics: ‘Therapy’, ‘harm’, ‘diagnosis’, ‘prognosis’, and ‘other’ had been investigated and published. Most studies (*N* = 62) were observational. Thirty-three prospective studies included between 61 and 12,022 patients, and 29 retrospective studies included between 57 and 3971 patients. Eleven were randomised controlled trials (50–452 patients), and four used a diagnostic accuracy design (124–803 patients). Two studies used qualitative methods (13 and 24 patients). We had planned to define additional topics in case several studies appeared with a study design we had not predefined. This was not the case since the group of ‘other’ was diverse and did not justify defining an additional topic.

Research questions regarding therapy and harm were mostly investigated in randomised controlled trials (11 out of 17 studies). Hydroxychloroquine was investigated in several studies and generally proved safe but ineffective against COVID-19 [9–12]. Two later studies investigated nitazoxanide and ivermectin, which both were ineffective [13,14]. Antibodies, antiandrogens, Peginterferon lambda, and proxalutamide, showed promising results on viral clearance or shedding [15–20]. No studies on therapy published before 1 April 2021 showed a clinically significant effect on outpatients with mild-moderate COVID-19.

Diagnostic questions were primarily investigated using an observational and not a diagnostic accuracy design (22 out of 26). Three of the diagnostic accuracy studies investigated saliva samples as an alternative to nasopharyngeal swabs, and although the initial study showed promising accuracy of saliva samples, later studies called for caution when used in outpatients with a low prevalence of COVID-19 [21–23]. A single, large diagnostic accuracy study including 803 healthcare workers attempted to identify a combination of symptoms, which could reliably identify COVID-19 positive patients in a separate cohort [24]. However, although some symptoms were highly correlated to the SARS-CoV-2 test result, the specificity of different combinations of symptoms remained low.

Prognostic research questions were investigated using both prospective and retrospective designs (10 and 11, respectively). Two large, prospective cohort studies, where patients with persistent symptoms were recruited from a Facebook group, reported high rates of care dependency and persistent symptoms three months after initial recovery [25,26]. Later studies on ‘long-COVID’ and persistent symptoms showed that dyspnoea and loss of smell and taste were most likely to persist and that long COVID was more likely with increasing age, body mass index, and female sex [27,28]. The distribution of topics and study designs are shown in Table 2.

**Table 2.** Topics and designs.

	Randomised controlled trial	Diagnostic accuracy	Observational–prospective	Observational–retrospective	Mixed-methods and qualitative	Total
Therapy	11	0	2	3	0	16
Harm	0	0	1	0	0	1
Diagnosis	0	4	14	8	0	26
Prognosis	0	0	10	11	0	21
Other	0	0	6	7	2	15
Total	11	4	33	29	2	79

The term ‘prospective’ was used for all studies where patients were enrolled and subjected to predefined procedures or data collection instruments. The term also includes cross-sectional studies with no prospective follow-up. The term ‘retrospective’ was used for all studies where data was based on historical clinical data collected from for example charts.

The importance of infection control in nursing homes was stressed in two studies from March and April 2020 evaluating outbreaks of COVID-19 in nursing homes in a single county in the USA [29,30]. High morbidity and mortality were found among the residents, and the potential for rapid transmission among residents, staff, and visitors at the facility was described. All included articles are listed in Supplementary Appendix 2.

## Discussion

### Main findings

We identified a number of studies relevant to general practice regarding symptomatic, non-hospitalised patients with mild to moderate suspected or confirmed COVID-19 disease. Only a few of these were conducted in general practice or other primary care settings, and the vast majority were on non-pregnant adults. Management of children, pregnant women, and frail elderly with suspected COVID-19 in general practice remains largely un-investigated.

### Strengths and limitations

This study is, to our knowledge, the first review of all published research regarding symptomatic, non-hospitalised patients with mild to moderate suspected or confirmed COVID-19 disease relevant to general practice. The systematic approach of the review made it possible to identify a substantial number of eligible studies, although identifying research from general practice can be challenging [7]. Since our search included search terms, such as ‘outpatients’ or ‘general practice’, we may have missed some studies from quarantine stations and testing facilities, but we did catch a number of these as well. The scientific method was rigorous and followed the PRISMA guideline for scoping reviews (Supplementary Appendix 3) [31]. However, several limitations are present. Our search was limited by the low number of identified search terms for COVID-19 when we started the review. Validated search strings have been developed since [32]. However, we tried to conduct our search with the updated search terms, which did not change the number of hits substantially. It was obvious from the search results that several countries have handled large groups of COVID-19 positive patients as in-patients in the early phases of the pandemic, and some of the research from those countries has thus not been eligible for inclusion in this review since it was difficult to apply these results to general practice. Therefore, for example, we could not identify any eligible studies on children because they were often admitted to the hospital in case of suspected COVID-19. Due to the lack of test capacity, especially in the early pandemic, we decided to include studies on both suspected and confirmed COVID-19. This leaves the possibility that some of the patients included in this review suffered from other infections than COVID-19.

### Relation to the existing literature

We identified a substantial number of studies on symptoms and their association to COVID-19. An observational study from Iceland including both symptomatic and asymptomatic patients confirmed that most PCR-positive COVID-19 patients had mild disease and that fever, headache, myalgia, and non-productive cough were prevalent symptoms at the debut of COVID-19 infection [33]. Headache, non-productive cough, dyspnoea, lethargy, and loss of taste and smell were the most common symptoms 14 days after the debut of COVID-19 infection. In our review, loss of taste and smell were heavily investigated and very prevalent symptoms in some studies. This indicates that patients may have been identified at a later stage of disease or that sampling of patients in those studies was not representative of the general population. This is well in line with the fact that several of these studies were investigating outpatients from ear-nose-throat departments. Systematic reviews on the predictive value of symptoms and signs have found little value of individual symptoms and signs in predicting COVID-19 and that the available evidence was poorly reported and at high risk of bias [34,35].

No convincing treatments for mild-to-moderate COVID-19 were identified as yet. A Cochrane review confirmed our findings that hydroxychloroquine had little or no effect on the risk of death in patients with COVID-19 in in-patients with more limited evidence for outpatients [36]. A ‘living systematic review’ has not identified any additional treatments with convincing clinical effects for outpatients [37]. A study from July 2021 in outpatients has shown a promising effect of inhaled budesonide on patient recovery [38].

In this review, we identified two early published studies describing the rapid transmission and fatal consequences of COVID-19 in nursing homes [29,30]. A national cross-sectional survey study from England investigating factors associated with SARS-CoV-2 infection and outbreaks in long-term care found that reduced transmission from staff to residents was associated with adequate sick pay for the staff, minimal use of agency staff, increased staff-to-bed ratio, and staff cohorting with either infected or uninfected residents [39]. Although general practitioners cannot change these organisational factors, it is important to be aware the busy nursing homes with a high staff turnover are also at a high risk of infection outbreaks.

### Implications

The included studies in this review were primarily observational with broad, descriptive aims and conducted in other places than general practice. The lack of research conducted in general practice could be due to lack of preparation, lack of funding, or lack of research ideas. Streamlined collaboration, preferably from strong organisations and primary care research networks, should be elaborated and committed to this kind of research. Although our findings do not allow for any strong conclusions on the clinical management of suspected COVID-19 patients in general practice, they do uncover a lack of relevant, high-quality evidence to guide diagnosing, triaging, and managing patients with suspected COVID-19 in general practice. Future studies should aim to include children, pregnant women, and frail elderly.

## Conclusion

Although GPs in most countries must have been involved in and affected by COVID-19, very little primary care research has been published on this topic during the first year of the pandemic. The treatment of children, pregnant women, and frail elderly suspected of COVID-19 remained largely unstudied in general practice

## Supplementary Material

Supplemental Appendix 3: PRISMA checklist

Supplemental Appendix 2: included articles

Supplemental Appendix 1: search string
