# Supplementary material for: Management of symptomatic patients with suspected mild-moderate COVID-19 in general practice. What was published within the first year of the pandemic? A scoping review
Source: Eur J Gen Pract. 2021 Nov 18;27(1):339–45. doi: 10.1080/13814788.2021.2002295 (PMC8604528; doi:10.1080/13814788.2021.2002295)
Supplement: Supplemental Appendix 2: included articles [file IGEN_A_2002295_SM1622.docx]

**Appendix 2: Included articles**

| **Topic** | **Date indexed in pubmed** | **Title** | **Country** | **Study setting** | **Research question** | **Main findings** |
| --- | --- | --- | --- | --- | --- | --- |
| Diagnosis | 22-04-2020 | Alterations in Smell or Taste in Mildly Symptomatic Outpatients With SARS-CoV-2 Infection [1] | Italy | Hospital (outpatient or staff) | We evaluated prevalence, intensity, and timing of an altered sense of smell or taste in patients with SARSCoV-2 infections. | Alterations in smell or taste were frequently reported by mildly symptomatic patients with SARS-CoV-2 infection and often were the first apparent symptom. |
| Diagnosis | 23-04-2020 | Strong associations and moderate predictive value of early symptoms for SARS-CoV-2 test positivity among healthcare workers, the Netherlands, March 2020 [2] | Netherlands | Hospital (outpatient or staff) | We aimed to identify symptoms associated with test positivity and develop a diagnostic model to predict SARS-CoV-2 infection based on early symptoms | Anosmia, muscle ache, ocular pain, general malaise, headache, extreme tiredness and fever were associated with positivity. A predictive model based on these symptoms showed moderate discriminative value (sensitivity: 91.2%;specificity: 55.6%). |
| Diagnosis | 28-04-2020 | COVID-19 anosmia reporting tool:initial findings.[3] | USA | Other primary care settings | To investigate the relationship of anosmia and dysgeusia to SARS-CoV2 infection (pilot) | Anosmia was noted in 73% of patients prior to COVID-19 diagnosis and was the initial symptom in 26.6%. Some improvement was noted in 27% of patients, with a mean time to improvement of 7.2 days in this group (85% of this group improved within 10 days) |
| Diagnosis | 01-05-2020 | Prevalence and Clinical Presentation of Health Care Workers With Symptoms of Coronavirus Disease 2019 in 2 Dutch Hospitals During an Early Phase of the Pandemic [4] | Netherlands | Hospital (outpatient or staff) | To determine the prevalence and clinical presentation of COVID-19 among HCWs with self-reported fever or respiratory symptoms | Within 2 weeks after the first Dutch case was detected, a substantial proportion of health care workers with self-reported fever or respiratory symptoms were infected with severe acute respiratory syndrome coronavirus 2. |
| Diagnosis | 01-05-2020 | Olfactory and Gustatory Dysfunction in Coronavirus Disease 2019 (COVID-19) [5] | Germany | Hospital (outpatient or staff) | To investigate prevalence of reduced olfaction and decreased sense of taste in European COVID-19 patients. | Olfactory and gustatory dysfunction are very common in COVID-19 patients, |
| Diagnosis | 03-05-2020 | Clinical characteristics of asymptomatic and symptomatic patients with mild COVID-19 [6] | South Korea | Quarantine station or testing site | To assess the prevalence of asymptomatic individuals in this outbreak and to characterize the symptoms of people with mild COVID-19. | We found that as much as one-fifth of individuals with COVID-19 had remained asymptomatic from potential exposure to laboratory confirmation and facility admission.Hyposmia was quite frequent among individuals with mild COVID-19, but fever was not. |
| Diagnosis | 11-05-2020 | Prevalence and Duration of Acute Loss of Smell or Taste in COVID-19 Patients [7] | Korea | Community | To determine the prevalence of acute loss of taste and smell and evaluate their diagnostic significance | Anosmia and ageusia seem to be part of important symptoms and clues for the diagnosis of COVID-19, particularly in the early stage of the disease. |
| Diagnosis | 15-05-2020 | Saliva sample as a non-invasive specimen for the diagnosis of coronavirus disease-2019 [8] | Thailand | Hospital (outpatient or staff) | We aimed to investigate the potential use of saliva samples as a non-invasive tool for the diagnosis of COVID-19 | The saliva-RT PCR test demonstrated high sensitivity and comparable performance to the current standard of nasopharyngeal and throat swab. |
| Diagnosis | 01-06-2020 | Association of chemosensory dysfunction and Covid-19 in patients presenting with influenza-like symptoms [9] | USA | Hospital (outpatient or staff) | To provide Insight into the timing and association of smell/taste loss and COVID-19 | In ambulatory individuals with influenza-like symptoms, chemosensory dysfunction was strongly associated with COVID-19 infection |
| Diagnosis | 23-06-2020 | Predictive factors of COVID-19 in patients with negative RT-qPCR [10] | Spain | Community | To evaluate the factors associated with false negatives in RT-qPCR in patients with mild-moderate symptoms of COVID-19. | Patients who present mild or moderate symptoms with negative RT-qPCR, but with fever and/or anosmia, should be considered assuspicious cases and should be evaluated with other diagnostic methods. |
| Diagnosis | 26-06-2020 | COVID-19 symptoms predictive of healthcare workers’ SARS-CoV-2 PCR results [11] | USA | Hospital (outpatient or staff) | To investigate the presenting symptoms most predictive of positive/negative SARS-CoV-2 RT-PCR results among health care workers | Anosmia/ageusia, fever, and myalgia were the strongest independent predictors of positive assays |
| Diagnosis | 01-07-2020 | Smell and Taste Symptom-Based Predictive Model for COVID-19 Diagnosis [12] | USA | Community | To identify a parsimonious subset of symptoms that would enable a clinically tractable classifier to predict COVID-19 positivity to improve both decision making on test ressource allocation and evidence-based counseling of concerned patients. | Smell or taste change is a strong predictor for a COVID-19 positive test result. Using the presence of smell or taste change with fever, this parsimonious classifier correctly predicts 75% of COVID-19 test results |
| Diagnosis | 18-07-2020 | Non-invasive saliva specimens for the diagnosis of COVID-19: caution in mild outpatient cohorts with low prevalence [13] | UK | Hospital (outpatient or staff) | To compare reverse transcription polymerase chain reaction (RTPCR)results from combined oropharyngeal/nasopharyngeal (OP/NP) swabs with saliva. | Rather than concluding saliva is a suitable alternative to naso-pharyngeal swab,we caution reliance on its use as a diagnostic test in this setting, without further robust evidence from a larger cohort of patients, to support its use. |
| Diagnosis | 31-07-2020 | Challenges in use of saliva for detection of SARS CoV-2 RNA in symptomatic outpatients [14] | USA | Quarantine station or testing site | To compare SARS CoV-2 positivity on paired naso-pharyngeal swab and saliva samples | Real-time RT-PCR of pure saliva had an overall sensitivity for SARS CoV-2 RNA detection of 85.7 % when compared to simultaneously collected naso-phagyngeal swab. |
| Diagnosis | 01-08-2020 | Acute smell and taste loss in outpatients: all infected with SARS-CoV-2? [15] | France | Hospital (outpatient or staff) | To compare the characteristics of loss of smell and taste between patients with a clinical diagnosis of SARS-CoV-2 infection to patients with a RT-PCR diagnosis. | this study showed that outpatients with loss of smell and taste with a clinical diagnosis had strong similarities with the RT-PCR proven SARSCoV-2 patients and should be considered positive. |
| Diagnosis | 07-08-2020 | Experience from a COVID-19 first-line referral clinic in Greater Copenhagen [16] | Denmark | Hospital (outpatient or staff) | To evaluate the symptom pattern of COVID-19 and the overlap with other upper respiratory tract infections | Specific symptoms of fever and dyspnoea were more often seen in patients with COVID-19 infection, whereas a sore throat was a negative predictor. |
| Diagnosis | 20-08-2020 | Epidemiological and clinical characteristics of SARS-CoV-2 infections at a testing site in Berlin [17] | Berlin | Quarantine station or testing site | To describe epidemiological and clinical characteristics and aim at identifying risk factors for SARS-CoV-2 detection during the first 6 weeks of operation | In this young population, early-onset presentation of COVID-19 resembled flu-like symptoms, except for smell and/or taste dysfunction. Risk factors for SARS-CoV-2 detection were return from regions with high incidence and contact with confirmed SARS-CoV-2 cases, particularly when tests were administered within the first 2 weeks after contact and/or onset of symptoms. |
| Diagnosis | 21-08-2020 | More than loss of taste and smell: burning watering eyes in coronavirus disease 2019 [18] | Germany | Hospital (outpatient or staff) | To evaluate ocular symptoms in European non-hospitalized patients with severe acute respiratory syndrome-related coronavirus 2 (SARS-CoV-2) and to investigate associations with the demographic data as well as nasal and general physical symptoms. | Ocular involvement in European non-hospitalized individuals with COVID-19 seems to be highly underestimated. Overall, these ocular symptoms, including burning sensations, epiphora and redness, seem to be mild and to not need treatment. |
| Diagnosis | 25-08-2020 | Chemosensory dysfunction in COVID-19 out-patients [19] | Spain | Hospital (outpatient or staff) | To investigate the frequency and severity of olfactory and gustatory dysfunction in SARS-CoV-2 (+) out-patients compared to controls with common cold/flu like symptoms and two negative RT-PCR. | Olfactory and gustatory dysfunction is a prevalent symptom in COVID-19 subjects with significant differences compared to controls. It was predominant in young and females subjects. |
| Diagnosis | 26-08-2020 | Diagnosis of COVID-19 Based on Symptomatic Analysis of Hospital Healthcare Workers in Belgium: Observational Study in a Large Belgian Tertiary Care Center during Early COVID-19 Outbreak [20] | Belgium | Hospital (outpatient or staff) | To identify early symptoms allowing rapid appraisal of infection with SARS-CoV-2 among healthcare workers of a large Belgian hospital | The presence of cough, myalgia, loss of smell/taste in combination with fever >37.5°C has a high positive predictive value for SARS-CoV-2 infection. |
| Diagnosis | 27-08-2020 | Usefulness of Lung Ultrasound Examinations Performed by Primary Care Physicians in Patients With Suspected COVID-19 [21] | Spain | General practice | To assess whether lung ultrasound findings in patients with suspected COVID-19 attended a PC center (without access to urgent chest x-ray or blood tests in the same center) to help physicians make better decisions about hospital referral. | The proposed lung ulstrasound severity scale was significantly associated with the main outcome of appropriate referral |
| Diagnosis | 10-09-2020 | Association of subjective olfactory dysfunction and 12-item odor identification testing in ambulatory COVID-19 patients [22] | USA | Hospital (outpatient or staff) | To estimate the correlation between self-reported and psychophysical olfactory function | Subjective olfactory assessment is useful in screening olfactory dysfunction at early disease time points when psychophysical testing cannot be conducted |
| Diagnosis | 03-11-2020 | Using Anti-SARS-CoV-2 IgG and IgM Antibodies to Detect Outpatient Cases with Olfactory and Taste Disorders Suspected as Mild Form of COVID-19: a Retrospective Survey [23] | Iran | Hospital (outpatient or staff) | To evaluate the COVID19 IgM and IgG titers in patients with olfactory and taste disorders | IgG antibody titers were higher in cases than in the control group. There was no correlation among antibody titers and the severity of olfactory disturbances, the gender, and the age. |
| Diagnosis | 16-02-2021 | Rate and predictive parameters of novel Coronavirus 2019 (Sars-CoV-2) infections in a German General Practice [24] | Germany | General practice | To investigate distribution of novel Coronavirus 2019 (SARS-CoV-2) infections in a German General Practice and to learn about possible predictive parameters regarding infection and pathways of transmission. | A total of 30% of patients had antibodies. It was not possible to identify one solid predictive symptom. |
| Diagnosis | 19-02-2021 | Validity of Clinical Symptoms Score to Discriminate Patients with COVID-19 from Common Cold Out-Patients in General Practitioner Clinics in Japan [25] | Japan | General practice | To investigate the clinical characteristics which discriminate COVID-19 by comparing between patients with and without COVID-19 among outpatients showing common cold symptoms who visited a general practitioner clinic | Anosmia, headache, sputum production, history of visiting an izakaya or bar were associated with COVID-19 |
| Diagnosis | 26-02-2021 | Positive ratio of polymerase chain reaction (PCR) and validity of pre-screening criteria at an outpatient screening center during the early phase of the COVID-19 epidemic in Japan [26] | Japan | Quarantine station or testing site | To evaluate COVID-19 positive ratio and pre-screening criteria in Tokyo immediately after insurance-covered SARS-CoV-2 polymerase chain reaction testing became available in Japan | The Ministry of Health, Labour and Welfare in Japan criteria, including symptoms and exposure/travel history, could support COVID-19 pre-screening. |
| Harm | 17-10-2020 | Hydroxychloroquine Safety Outcome within Approved Therapeutic Protocol for COVID-19 Outpatients in Saudi Arabia [27] | Saudi Arabia | Hospital (outpatient or staff) | To assess the safety outcomes and reported adverse events among COVID-19 patients attending outpatient fever clinics and subjected to the approved treatment protocol within 3–7 days in Saudi Arabia | Hydroxychloroquine use for COVID-19 patients with mild to moderate symptoms inanoutpatient settingwiththe recommendedprotocol andinclusion/exclusioncriteria is safe, is highly tolerable and has minimal side effects |
| Other | 27-03-2020 | Epidemiology of Covid-19 in a Long-Term Care Facility in King County, Washington [42] | USA | Other primary care settings | To descibe an outbreake of COVID-19 in a long-term care facility | Outbreaks of Covid-19 in long-term care facilities can have a considerable impact on vulnerable older adults and local health care systems. The findings also suggest that once Covid-19 has been introduced into a long-term care facility, it has the potential to spread rapidly and widely |
| Other | 24-04-2020 | Presymptomatic SARS-CoV-2 Infections and Transmission in a Skilled Nursing Facility [28] | USA | Other primary care settings | To assess the extent of transmission and to evaluate the adequacy of symptom-based screening of residents to identify infections. | Rapid and widespread transmission of SARS-CoV-2 was demonstrated in this skilled nursing facility. More than half of residents with positive test results were asymptomatic at the time of testing and most likely contributed to transmission. |
| Other | 30-05-2020 | Clinical features of 1487 COVID-19 patients with outpatient management in the Greater Paris: the COVID-call study [29] | France | Hospital (outpatient or staff) | To describe the clinical characteristics of outpatients with mild to moderat COVID-19 | Digestive and ear–nose–throat symptoms were frequent. |
| Other | 17-06-2020 | Clinical and Epidemiological Characteristics of 1,420 European Patients with mild-to-moderate Coronavirus Disease 2019. [30] | European countries | Hospital (outpatient or staff) | To study the clinical presentation of Covid-19 in Europe. | The clinical presentation of mild-to-moderate Covid-19 substantially varies according to the age and the sex characteristics of patients. Olfactory dysfunction seems to be an important underestimated symptom of mild-to-moderate Covid-19 that needs to be recognized as such by the WHO. |
| Other | 02-07-2020 | COVID-19 in health-care workers in three hospitals in the south of the Netherlands: a cross-sectional study [31] | Netherlands | Hospital (outpatient or staff) | We aimed to gain insight in possible sources of infection in health-care workers. | Although direct transmission in the hospitals cannot be ruled out, our data do not support widespread nosocomial transmission as the source of infection in patients or health-care workers. |
| Other | 04-07-2020 | COVID- 19 infection: strategies on when to discontinue isolation, a retrospective study [32] | USA | Hospital (outpatient or staff) | To help develop additional, more conservative guidelines for discontinuation of self-isolation that providers may consider having their patients follow for self-isolation discontinuation. | 53% of patients still showed detectable viral RNA despite meeting CDC guidelines for discontinuation of self-isolation |
| Other | 12-08-2020 | Investigation and Analysis of 108 Cases of Home Isolated Patients with Mild COVID-19 [33] | China | Community | To evaluate participants’ compliance with various epidemic prevention measures | During a period of home isolation under the guidance of a doctor, individuals can comply with epidemic prevention measures and symptoms can be improved |
| Other | 14-08-2020 | Non-hospitalized Adults with COVID-19 Differ Noticeably from Hospitalized Adults in Their Demographic, Clinical, and Social Characteristics [34] | USA | Hospital (outpatient or staff) | To provide a description of the demographics, comorbidities, clinical presentation, and social factors in confirmed SARS-CoV-2-positive non-hospitalized adults | Our results suggest the demographic and clinical characteristics of COVID-19 illness in non-hospitalized adults differ considerably from hospitalized patients |
| Other | 05-09-2020 | Association of contact to small children with a mild course of COVID-19 [35] | Germany | Community | If a childhood-related infection were protective against a severe course of COVID-19, it would be expected that adults with intensive and regular contact with small children also may have a mild course of COVID-19 more frequently | In the relatively small subgroup with intensive care treatment, patients without contact with small children were overrepresented. Again, this is not well explained by age, gender, or BMI distribution for this subgroup. |
| Other | 11-09-2020 | Community seroprevalence of COVID-19 in probable and possible cases at primary health care centres in Spain [36] | Spain | General practice | To measure the seroprevalence of antibodies against SARS-CoV-2 infection in a community sample of possible cases and among probable cases followed in primary care | The seroprevalence of antibodies against SARS-CoV-2 among possible cases was lower than expected. Approximately, 40% of the symptomatic patients followed up by GPs duringthe peak months of the pandemic were positive. |
| Other | 09-12-2020 | Generic and Respiratory-Specific Quality of Life in Non-Hospitalized Patients with COVID-19 [37] | Belgium | Community | To assess respiratory-specific quality of life in addition to generic quality of life in former patients with confirmed/suspected COVID-19 who have never been admitted to the hospital. | Both generic and respiratory-specific quality of life are affected in non-hospitalized patients with COVID-19, approximately three months after the onset of symptoms. |
| Other | 15-12-2020 | Finding the 'right' GP: a qualitative study of the experiences of people with long-COVID [38] | UK | Community | To explore experiences of people with persisting symptoms following COVID-19 infection, and their views on primary care support received. | Patients require their GP to believe their symptoms and to demonstrate empathy and understanding. Ongoing support by primary care professionals during recovery and rehabilitation is crucial. |
| Other | 17-12-2020 | Prevalence of COVID-19 infection and outcomes among symptomatic healthcare workers in Seattle, Washington [39] | USA | Hospital (outpatient or staff) | To determine the prevalence of infections among symptomatic frontline vs nonfrontline staff, and clinical outcomes associated with COVID-19 in these employees | During the study period, we observed that the prevalence of positive SARS-CoV-2 tests among symptomatic health care workers was comparable to that of symptomatic nonfrontline staff |
| Other | 19-12-2020 | Clinical learnings from a virtual primary care program monitoring mild to moderate COVID-19 patients at home [40] | Canada | General practice | To describe the natural history, clinical management and outcomes of patients who received care during the first 5 weeks of the CovidCare@Home virtual clinic. | Patients with mild to moderate COVID-19 disease can be managed safely and effectively in a family medicine-led virtual program. Some sex differences in symptoms were observed. |
| Other | 22-03-2021 | Reluctant pioneer': A qualitative study of doctors' experiences as patients with long COVID [41] | UK | Community | To report the experiences of doctors with long COVID. | Experiencing long COVID can be transformative: many expressed hope that good would come of their experiences. Distress related to feelings of being ‘let down’ and the hard work of trying to access care. Participants highlighted that they felt better able to care for, and empathize with, patients with chronic conditions, particularly where symptoms are unexplained |
| Prognosis | 21-05-2020 | COVID-19 in pregnant women: case series from one large New York City obstetrical practice [43] | USA | Other primary care settings | To report a case series of pregnant women in New York City with confirmed or presumed coronavirus disease (COVID-19) infection | Among 92 women with confirmed or presumed COVID-19, the overall morbidity was low |
| Prognosis | 07-06-2020 | Self-reported olfactory loss associates with outpatient clinical course in COVID-19 [44] | USA | Hospital (outpatient or staff) | To investigate the association between selfreported anosmia and hospital admission during the course of Covid-19. | Normosmia is an independent predictor of admission in Covid-19 cases. Smell loss in Covid-19 may associate with a milder clinical course |
| Prognosis | 31-07-2020 | Serologic responses to SARS-CoV-2 infection among hospital staff with mild disease in eastern France [45] | France | Hospital (outpatient or staff) | To assess antibody kinetics in individuals who had recovered from COVID-19 and to understand how this correlates with protective immunity. | Antibodies against SARS-CoV-2 were detected in virtually all hospital staff sampled from 13 days after the onset of COVID-19 symptoms |
| Prognosis | 01-08-2020 | Symptoms and critical illness among obstetric patients with coronavirus disease 2019 (COVID-19) infection [46] | USA | Hospital (outpatient or staff) | To characterize symptoms and disease severity among pregnant women with coronavirus disease 2019 (COVID-19) infection, along with laboratory findings, imaging, and clinical outcomes. | Of 158 pregnant women with COVID-19 infection, 124 (78%) had mild or asymptomatic disease and 34 (22%) had moderate or severe disease. |
| Prognosis | 01-09-2020 | Chemosensory dysfunction in COVID-19: prevalences, recovery rates, and clinical associations on a large brazilian sample [47] | Brazil | Community | To measure the percentage of reported olfactory or taste losses and their severity, recovery time, and association with other features in a large cohort of patients with COVID-19. | The prevalence of self-reported chemosensory dysfunction is high among patients with COVID-19. Almost all patients seem to recover a significant part of their smell and taste abilities in the first 4 months after the onset of symptoms. |
| Prognosis | 12-09-2020 | Care Dependency in Non-Hospitalized Patients with COVID-19 [48] | Netherlands | Community | To explore the level of care dependency and the need for assistance with personal care in non-hospitalized COVID-19 patients. | COVID-19 has an important impact on care dependency in non-hospitalized patients. About three months after the onset of symptoms, a considerable proportion of non-hospitalized patients were to some degree dependent on others for personal care. |
| Prognosis | 26-09-2020 | Does phenotypic expression of bitter taste receptor T2R38 show association with COVID-19 severity? [49] | USA | Hospital (outpatient or staff) | We assessed a potential association between phenotypic expression of T2R38 and outcome of COVID-19, with special attention to clinical course requiring hospitalization. | Phenotypic expression of T2R38 with taste strip testing appears to associate with the clinical course and symptomatology specific to each individual as 100% of the patients requiring inpatient admission were classified as non-tasters. |
| Prognosis | 28-09-2020 | Emergence and Evolution of Olfactory and Gustatory Symptoms in Patients with COVID-19 in the Outpatient Setting [50] | Iran | Hospital (outpatient or staff) | To evaluate the prevalence of general and sinonasal symptoms in patients with olfactory symptoms and mild coronavirus disease-2019 (COVID-19) and determining the patterns in emergence and resolution of olfactory/gustatory symptoms relative to general and sinonassal symptoms. | Our study showed that hyposmia and anosmia in mild COVID-19 are frequently associated with general and sinonasal symptoms and tend to persist longer than the general and sinonasal symptoms during the course of the disease. |
| Prognosis | 26-10-2020 | Persistent symptoms 3 months after a SARS-CoV-2 infection: the post-COVID-19 syndrome? [51] | Netherlands | Community | This study assessed whether or not multiple relevant symptoms recover following the onset of symptoms in hospitalised and nonhospitalised patients with COVID-19 | There is only a partial recovery in symptoms about 3 months after the onset of symptoms |
| Prognosis | 27-10-2020 | Protocol-Driven Intensive Outpatient Management of Pregnant Patients With Symptomatic Coronavirus Disease 2019 [52] | USA | Hospital (outpatient or staff) | To describe the natural history of COVID-19 infection in symptomatic pregnant women | Management of the majority of pregnant women with symptomatic COVID-19 illness can be accomplished in the outpatient setting |
| Prognosis | 02-11-2020 | Pregnancy Outcomes Among Women With and Without Severe Acute Respiratory Syndrome Coronavirus 2 Infection [53] | USA | Hospital (outpatient or staff) | To evaluate adverse outcomes associated with severe acute respiratory syndrome coronavirus 2 (SARS-CoV-2) infection in pregnancy and to describe clinical management, disease progression, hospital admission, placental abnormalities, and neonatal outcomes. | SARS-CoV-2 infection during pregnancy was not associated with adverse pregnancy outcomes. Neonatal infection may be as high as 3% and may occur predominantly among asymptomatic or mildly symptomatic women. Among 132 women with mild symptomatic infection, 126 remained mild |
| Prognosis | 20-11-2020 | COVID-19 symptoms, duration, and prevalence among healthcare workers in the New York metropolitan area [54] | USA | Hospital (outpatient or staff) | To examine the relationships between symptoms of COVID-19 and both PCR and IgG antibody test results, as well as between PCR positivity and IgG antibody detection | Health care workers with COVID-19 who did not require hospitalization still had prolonged illness. Shortness of breath, fever, sore throat, and diarrhea are associated with longer durations of time away from work |
| Prognosis | 30-11-2020 | Long COVID in the Faroe Islands - a longitudinal study among non-hospitalized patients [55] | Denmark (Faroe Islands) | Community | To describe symptoms in the acute phase and especially long COVID in mainly non-hospitalized patients from the Faroe Islands | Our results show that it might take months for symptoms to resolve, even among nonhospitalized persons with mild illness course in the acute phase |
| Prognosis | 08-12-2020 | COVID-19 Symptoms: Longitudinal Evolution and Persistence in Outpatient Settings [56] | Schwitzerland | Quarantine station or testing site | To describe COVID-19 symptom evolution and persistence in an outpatient setting in Geneva, Switzerland, from day 1 through day 30 to 45 after diagnosis. | Our study shows persistence of symptoms in a third of ambulatory patients 30 to 45 days after diagnosis fatigue, dyspnea, and loss of taste or smell were the main persistent symptoms. |
| Prognosis | 29-12-2020 | Wide spectrum of clinical picture of COVID-19 in children - From mild to severe disease [57] | Poland | Hospital (outpatient or staff) | To analyse the frequency, clinical picture and outcome of COVID-19 in children based on the experience from the tertiary care centre and regional sanitary-epidemiological office. | In general, the clinical course of COVID-19 was mild with anosmia and dysgeusia as the most common symptoms. However, in hospitalised children, a severe progression ofthe disease and less typical signs as aplastic anaemia may be developed |
| Prognosis | 01-01-2021 | Olfactory dysfunction in COVID-19: a marker of good prognosis [58] | Brazil | Hospital (outpatient or staff) | To assess the association between olfactory disorders in patients with COVID-19 and the severity of the flu syndrome (mild flu syndrome, severe flu syndrome, critical illness). | Olfaction dysfunction was significantly more prevalent in patients with mild flu syndrome in COVID-19. It may be a predictor of a good prognosis for this infection |
| Prognosis | 06-01-2021 | Symptoms and recovery among adult outpatients with and without COVID-19 at 11 healthcare facilities-July 2020, United States [59] | USA | Hospital (outpatient or staff) | To compare symptom prevalence and recovery among adults with and without COVID-19 who were tested at outpatient health facilities for SARS-CoV-2 infection during July 2020 | Differentiating COVID-19 from other acute illnesses will require widespread diagnostic testing, especially during influenza seasons. Persistent COVID-19- related symptoms may negatively affect quality of life, even among those initially presenting with mild illness. |
| Prognosis | 14-01-2021 | Symptoms and Clinical Outcomes of Coronavirus Disease 2019 in the Outpatient Setting [60] | USA | Hospital (outpatient or staff) | To describe the symptom profile and associated outcomes in a virtual outpatient COVID-19 clinic | We found a higher incidence of sinus symptoms, gastrointestinal symptoms, and myalgia and a lower incidence of fever, anosmia, and ageusia among our mild/moderate cases. Asthma and immunocompromised status were associated with adverse outcomes, and asthma and early symptoms of ageusia or myalgia with significantly longer duration of viral shedding. |
| Prognosis | 05-02-2021 | Treatment, Persistent Symptoms, and Depression in People Infected with COVID-19 in Bangladesh [61] | Bangladesh | Community | To examine treatment, persistent symptoms, and depression in people who had been infected with COVID-19 in Bangladesh | More than 50% of respondents reported that their health was not good, with sleep disturbances and difficulties engaging in physical activity |
| Prognosis | 21-03-2021 | Attributes and predictors of long COVID [62] | UK | Community | To investigate whether it is possible to predict a protracted course early in the disease. | Long COVID was more likely with increasing age and body mass index and female sex. Experiencing more than five symptoms during the first week of illness was associated with long COVID |
| Prognosis | 26-03-2021 | Factors associated with SARS-CoV-2 infection in patients attending an acute hospital ambulatory assessment unit [63] | Ireland | Hospital (outpatient or staff) | To describe the presenting features of patients with mild‐to‐moderate symptoms who met clinical criteria for suspected SARS‐CoV‐2, predictive symptoms, and factors affecting hospitalization. | Female patients were more likely to be hospitalized (p = .01) as were current and ex‐smokers (p = .05). “Non‐detectable” patients remain likely to require prolonged hospitalization. |
| Therapy | 16-07-2020 | Hydroxychloroquine for Early Treatment of Adults with Mild Covid-19: A Randomized-Controlled Trial [64] | Spain | Community | We assessed the efficacy and safety of hydroxychloroquine initiated early for treating outpatients with mild Covid-19 using the WHO core outcome set. | Compared with usual care, early treatment with hydroxychloroquine failed to reduce the RNA viral load in nasopharyngeal swabs after 3 and 7 days of treatment and shorten the time to complete resolution of symptoms in adults with PCR-confirmed mild Covid-19. |
| Therapy | 16-07-2020 | Hydroxychloroquine in Nonhospitalized Adults With Early COVID-19: A Randomized Trial [65] | USA | Community | To investigate whether hydroxychloroquine could reduce COVID-19 severity in adult outpatients. | Hydroxychloroquine did not substantially reduce symptom severity in outpatients with early, mild COVID-19 |
| Therapy | 10-08-2020 | Hanshiyi Formula, a medicine for Sars-CoV2 infection in China, reduced the proportion of mild and moderate COVID-19 patients turning to severe status: A cohort study [66] | China | Quarantine station or testing site | We aimed to evaluate the effect of Hanshiyi Formula on the progression to severe disease in mild and moderate COVID-19 patients | Hanshiyi Formula can significantly reduce the progression to severe disease in patients with mild and moderate COVID-19, which may effectively prevent and treat the disease. |
| Therapy | 01-12-2020 | COVID-19 outpatients: early risk-stratified treatment with zinc plus low-dose hydroxychloroquine and azithromycin: a retrospective case series studyycin: a retrospective case series study. [67] | USA | General practice | To show whether (i) a simple-to-perform outpatient risk stratification might allow for a rapid treatment decision shortly after onset of symptoms and (ii) whether the 5-day triple therapy with zinc, low-dose hydroxychloroquine and azithromycin might result in fewer hospitalisations and fatalities compared with relevant public reference data of untreated patients. | Risk stratification-based treatment of COVID-19 outpatients as early as possible after symptom onset using triple therapy, including the combination of zinc with low-dose hydroxychloroquine, was associated with significantly fewer hospitalisations. |
| Therapy | 09-12-2020 | Efficacy and safety of hydroxychloroquine in healthcare professionals with mild SARS-CoV-2 infection: Prospective, non-randomized trial [68] | Spain | Hospital (outpatient or staff) | To assess whether treatment with hydroxychloroquine alone reduces the time to negative PCR, and the symptoms of healthcare personnel with mild illness by COVID-19, who did not require initial hospitalization | Our study failed to show a substantial benefit of hydroxychloroquine in viral dynamics and in resolution of clinical symptoms in health care workers with mild COVID-19. |
| Therapy | 11-12-2020 | Safety and efficacy of hydroxychloroquine in 152 outpatients with confirmed COVID-19: A pilot observational study [69] | Turkey | Hospital (outpatient or staff) | To assess the efficacy and adverse reactions of hydroxychloroquine in ambulatory patients with COVID-19 admitted to the emergency department | Hydroxychloroquine is safe for COVID-19 and not associated with a risk of ventricular arrhythmia due to drug-induced QTc interval prolongation. |
| Therapy | 30-12-2020 | Clinical outcomes after early ambulatory multidrug therapy for high-risk SARS-CoV-2 (COVID-19) infection [70] | USA | Hospital (outpatient or staff) | This report discloses real world data and the clinical outcomes of early ambulatory treatment of acute COVID-19 in patients at high risk for hospitalization and death. | Empiric multidrug treatment for ambulatory COVID-19 according to age, comorbidities, and initial severity of symptoms is feasible with close follow-up. |
| Therapy | 14-01-2021 | Early use of nitazoxanide in mild Covid-19 disease: randomised, placebo-controlled trial [71] | Brazil | Hospital (outpatient or staff) | To evaluate whether early nitazoxanide therapy would be effective in accelerating symptom resolution | In patients with mild Covid-19, symptom resolution did not differ between nitazoxanide and placebo groups after 5 days of therapy. However, early nitazoxanide therapy was safe and reduced viral load significantly. |
| Therapy | 21-01-2021 | SARS-CoV-2 neutralizing antibody LY-CoV555 in outpatients with COVID-19 [72] | USA | Hospital (outpatient or staff) | To evaluate the efficacy and safety of LY-CoV555 in patients with recently diagnosed mild or moderate Covid-19 in the outpatient setting | By day 11, the majority of patients had a substantial trend toward viral clearance, including those in the placebo group. At day 29, the percentage of patients who were hospitalized with Covid-19 was 1.6% (5 of 309 patients) in the LY-CoV555 group and 6.3% (9 of 143 patients) in the placebo group. |
| Therapy | 21-01-2021 | REGN-COV2, a neutralizing antibody cocktail, in outpatients with COVID-19 [73] | USA | Hospital (outpatient or staff) | To investigate two fully human, neutralizing monoclonal antibodies against severe acute respiratory syndrome coronavirus 2 (SARS-CoV-2) spike protein, used in a combined cocktail (REGN-COV2) to reduce the risk of the emergence of treatment-resistant mutant virus. | The REGN-COV2 antibody cocktail reduced viral load, with a greater effect in patients whose immune response had not yet been initiated or who had a high viral load at baseline. |
| Therapy | 01-02-2021 | Early Antiandrogen Therapy With Dutasteride Reduces Viral Shedding, Inflammatory Responses, and Time-to-Remission in Males With COVID-19: A Randomized, Double-Blind, Placebo-Controlled Interventional Trial (EAT-DUTA AndroCoV Trial – Biochemical [74] | Brazil | Community | To determine the potential benefit of dutasteride, a commonly used broad and potent 5ARi, as a treatment for COVID-19. | The findings from this study suggest that in males with mild COVID-19 symptoms undergoing early therapy with nitazoxanide and azithromycin, treatment with dutasteride reduces viral shedding and inflammatory markers compared to males treated with a placebo. |
| Therapy | 05-02-2021 | Peginterferon lambda for the treatment of outpatients with COVID-19: a phase 2, placebo-controlled randomised trial [75] | Canada | Quarantine station or testing site | To investigate the safety and efficacy of peginterferon lambda in the treatment of outpatients with mild-to-moderate COVID-19. | Peginterferon lambda accelerated viral decline in outpatients with COVID-19, increasing the proportion of patients with viral clearance by day 7, particularly in those with high baseline viral load |
| Therapy | 22-02-2021 | Proxalutamide Significantly Accelerates Viral Clearance and Reduces Time to Clinical Remission in Patients with Mild to Moderate COVID-19: Results from a Randomized, Double-Blinded, Placebo-Controlled Trial [76] | Brazil | Community | To test whether the androgen receptor antagonist, Proxalutamide, would be a beneficial treatment for subjects with SARS-CoV-2 infection. | Proxalutamide significantly accelerated viral clearance on Day 7 in mild to moderate COVID-19 patients versus placebo |
| Therapy | 04-03-2021 | Effect of Ivermectin on Time to Resolution of Symptoms Among Adults With Mild COVID-19: A Randomized Clinical Trial [77] | Colombia | Community | To determine whether ivermectin is an efficacious treatment for mild COVID-19 | The findings do not support the use of ivermectin for treatment of mild COVID-19 |
| Therapy | 21-03-2021 | Efficacy and safety of Levamisole treatment in clinical presentations of non-hospitalized patients with COVID-19: a double-blind, randomized, controlled trial [78] | Iran | Hospital (outpatient or staff) | To evaluate the efficacy and safety of Levamisole when compared to the routine standard of care in non-hospitalized patients with mild to moderate COVID-19. | The results of the current study suggest that Levamisole may improve most of clinical status of patients with COVID-19. The patients receiving Levamisole had significantly better chance of clinical status including cough and dyspnea on day 14 when compared to the placebo. However, the effect-size of this finding has uncertain clinical importance. |
| Therapy | 30-03-2021 | Peginterferon Lambda-1a for treatment of outpatients with uncomplicated COVID-19: a randomized placebo-controlled trial [79] | USA | Community | To investigate if a single, 180mcg subcutaneous injection of Lambda would be associated with a shorter duration of viral shedding in comparison to a normal saline placebo injection. | A single subcutaneous injection of Lambda in outpatients with uncomplicated SARS-CoV-2 infection did not significantly reduce time to viral clearance or resolution of symptoms compared with placebo. |

References:

1 Spinato G, Fabbris C, Polesel J, *et al.* Alterations in Smell or Taste in Mildly Symptomatic Outpatients with SARS-CoV-2 Infection. *JAMA - J Am Med Assoc* 2020;**323**:2089–91. doi:10.1001/jama.2020.6771

2 Tostmann A, Bradley J, Bousema T, *et al.* Strong associations and moderate predictive value of early symptoms for SARS-CoV-2 test positivity among healthcare workers, the Netherlands, March 2020. *Eurosurveillance* 2020;**25**. doi:10.2807/1560-7917.ES.2020.25.16.2000508

3 Kaye R, Chang CWD, Kazahaya K, *et al.* COVID-19 Anosmia Reporting Tool: Initial Findings. *Otolaryngol - Head Neck Surg (United States)* 2020;**163**:132–4. doi:10.1177/0194599820922992

4 Kluytmans-van den Bergh MFQ, Buiting AGM, Pas SD, *et al.* Prevalence and Clinical Presentation of Health Care Workers With Symptoms of Coronavirus Disease 2019 in 2 Dutch Hospitals During an Early Phase of the Pandemic. *JAMA Netw open* 2020;**3**:e209673. doi:10.1001/jamanetworkopen.2020.9673

5 Luers JC, Rokohl AC, Loreck N, *et al.* Olfactory and Gustatory Dysfunction in Coronavirus Disease 2019 (COVID-19). *Clin Infect Dis* 2020;**71**:2262–4. doi:10.1093/cid/ciaa525

6 Kim J, Kim J, Wang Y. Clinical characteristics of asymptomatic and symptomatic patients with mild COVID-19. *Clin Microbiol Infect* 2020;**26**.

7 Lee Y, Min P, Lee S, *et al.* Prevalence and duration of acute loss of smell or taste in COVID-19 patients. *J Korean Med Sci* 2020;**35**:1–6. doi:10.3346/JKMS.2020.35.E174

8 Pasomsub E, Watcharananan SP, Boonyawat K, *et al.* Saliva sample as a non-invasive specimen for the diagnosis of coronavirus disease 2019: a cross-sectional study. *Clin Microbiol Infect* Published Online First: 2020. doi:10.1016/j.cmi.2020.05.001

9 Yan CH, Faraji F, Prajapati DP, *et al.* Association of chemosensory dysfunction and COVID-19 in patients presenting with influenza-like symptoms. *Int Forum Allergy Rhinol* 2020;**10**:806–13. doi:10.1002/alr.22579

10 López de la Iglesia J, Fernández-Villa T, Rivero A, *et al.* Predictive factors of COVID-19 in patients with negative RT-qPCR. *Semergen* 2020;**46**:6–11. doi:10.1016/j.semerg.2020.06.010

11 Lan FY, Filler R, Mathew S, *et al.* COVID-19 symptoms predictive of healthcare workers’ SARS-CoV-2 PCR results. *PLoS One* 2020;**15**:1–12. doi:10.1371/journal.pone.0235460

12 Roland LT, Gurrola JG, Loftus PA, *et al.* Smell and taste symptom-based predictive model for COVID-19 diagnosis. *Int Forum Allergy Rhinol* 2020;**10**:832–8. doi:10.1002/alr.22602

13 Skolimowska K, Rayment M, Jones R, *et al.* Non-invasive saliva specimens for the diagnosis of COVID-19: caution in mild outpatient cohorts with low prevalence. *Clin Microbiol Infect* 2020;**26**:1711–3. doi:10.1016/j.cmi.2020.07.015

14 Landry ML, Criscuolo J, Peaper DR. Challenges in use of saliva for detection of SARS CoV-2 RNA in symptomatic outpatients. *J Clin Virol* 2020;**130**:19–22. doi:10.1016/j.jcv.2020.104567

15 Renaud M, Leon A, Trau G, *et al.* Acute smell and taste loss in outpatients: All infected with SARS-CoV-2? *Rhinology* 2020;**58**:1–6. doi:10.4193/Rhin20.199

16 Kronborg TM, Kimer N, Junker AE, *et al.* Experience from a covid-19 first-line referral clinic in greater Copenhagen. *Dan Med J* 2020;**67**:1–10.

17 Maechler F, Gertler M, Hermes J, *et al.* Epidemiological and clinical characteristics of SARS-CoV-2 infections at a testing site in Berlin, Germany, March and April 2020—a cross-sectional study. *Clin Microbiol Infect* 2020;**26**:1685.e7-1685.e12. doi:10.1016/j.cmi.2020.08.017

18 Rokohl AC, Loreck N, Wawer Matos PA, *et al.* More than loss of taste and smell: burning watering eyes in coronavirus disease 2019. *Clin Microbiol Infect* 2020;**26**:1560.e5-1560.e8. doi:10.1016/j.cmi.2020.08.018

19 Rojas-Lechuga MJ, Izquierdo-Domínguez A, Chiesa-Estomba C, *et al.* Chemosensory dysfunction in COVID-19 out-patients. *Eur Arch Oto-Rhino-Laryngology* Published Online First: 2020. doi:10.1007/s00405-020-06266-3

20 Van Loon N, Verbrugghe M, Cartuyvels R, *et al.* Diagnosis of COVID-19 Based on Symptomatic Analysis of Hospital Healthcare Workers in Belgium. *J Occup Environ Med* 2020;**Publish Ah**. doi:10.1097/jom.0000000000002015

21 Calvo-Cebrián A, Alonso-Roca R, Rodriguez-Contreras FJ, *et al.* Usefulness of Lung Ultrasound Examinations Performed by Primary Care Physicians in Patients With Suspected COVID-19. *J Ultrasound Med* 2020;:1–10. doi:10.1002/jum.15444

22 Prajapati DP, Shahrvini B, MacDonald B V., *et al.* Association of subjective olfactory dysfunction and 12-item odor identification testing in ambulatory COVID-19 patients. *Int Forum Allergy Rhinol* 2020;**10**:1209–17. doi:10.1002/alr.22688

23 Taziki Balajelini MH, Vakili MA, Saeidi M, *et al.* Using Anti-SARS-CoV-2 IgG and IgM Antibodies to Detect Outpatient Cases with Olfactory and Taste Disorders Suspected as Mild Form of COVID-19: a Retrospective Survey. *SN Compr Clin Med* 2020;**2**:2554–60. doi:10.1007/s42399-020-00623-3

24 Paar M, Strumann C, Giesen H. Rate and predictive parameters of novel Coronavirus 2019 (Sars-CoV-2) infections in a German General Practice. *Ir J Med Sci* 2021;**2019**. doi:10.1007/s11845-021-02555-w

25 Sonoda S, Kuramochi J, Matsuyama Y, *et al.* Validity of Clinical Symptoms Score to Discriminate Patients with COVID-19 from Common Cold Out-Patients in General Practitioner Clinics in Japan. *J Clin Med* 2021;**10**. doi:10.3390/jcm10040854

26 Ide S, Hayakawa K, Yamamoto K, *et al.* Positive ratio of polymerase chain reaction (PCR) and validity of pre-screening criteria at an outpatient screening center during the early phase of the COVID-19 epidemic in Japan. Jpn. J. Infect. Dis. 2021. doi:10.7883/yoken.jjid.2020.813

27 Mohana A, Sulaiman T, Mahmoud N, *et al.* Hydroxychloroquine Safety Outcome within Approved Therapeutic Protocol for Covid-19 Outpatients in Saudi Arabia. *Int J Infect Dis* Published Online First: 2020. doi:10.1101/2020.08.16.20175752

28 Arons MM, Hatfield KM, Reddy SC, *et al.* Presymptomatic SARS-CoV-2 Infections and Transmission in a Skilled Nursing Facility. *N Engl J Med* 2020;**382**:2081–90. doi:10.1056/nejmoa2008457

29 Lapostolle F, Schneider E, Vianu I, *et al.* Clinical features of 1487 COVID-19 patients with outpatient management in the Greater Paris: the COVID-call study. *Intern Emerg Med* 2020;**15**:813–7. doi:10.1007/s11739-020-02379-z

30 Lechien JR, Chiesa-Estomba CM, Place S, *et al.* Clinical and epidemiological characteristics of 1420 European patients with mild-to-moderate coronavirus disease 2019. *J Intern Med* 2020;**288**:335–44. doi:10.1111/joim.13089

31 Sikkema RS, Pas SD, Nieuwenhuijse DF, *et al.* COVID-19 in health-care workers in three hospitals in the south of the Netherlands: a cross-sectional study. *Lancet Infect Dis* 2020;**20**:1273–80. doi:10.1016/S1473-3099(20)30527-2

32 Woodruff A, Walsh KL, Knight D, *et al.* COVID-19 infection: Strategies on when to discontinue isolation, a retrospective study. *Am J Infect Control* 2020;**48**:1032–6. doi:10.1016/j.ajic.2020.06.220

33 Li H, Peng YY, Lu JP. Investigation and Analysis of 108 Cases of Home Isolated Patients with Mild COVID-19. *Disaster Med Public Health Prep* 2020;:13–6. doi:10.1017/dmp.2020.296

34 Bergquist SH, Partin C, Roberts DL, *et al.* Non-hospitalized Adults with COVID-19 Differ Noticeably from Hospitalized Adults in Their Demographic, Clinical, and Social Characteristics. *SN Compr Clin Med* 2020;**2**:1349–57. doi:10.1007/s42399-020-00453-3

35 Dugas M, Schrempf IM, Ochs K, *et al.* Association of contact to small children with a mild course of COVID-19. *Int J Infect Dis* 2020;**100**:314–5. doi:10.1016/j.ijid.2020.09.003

36 Montenegro P, Brotons C, Serrano J, *et al.* Community seroprevalence of COVID-19 in probable and possible cases at primary health care centres in Spain. *Fam Pract* 2020;:1–6. doi:10.1093/fampra/cmaa096

37 Meys R, Delbressine JM, Goërtz YMJ, *et al.* Generic and Respiratory-Specific Quality of Life in Non-Hospitalized Patients with COVID-19. *J Clin Med* 2020;**9**:3993. doi:10.3390/jcm9123993

38 Kingstone T, Taylor AK, O’Donnell CA, *et al.* Finding the ‘right’ GP: a qualitative study of the experiences of people with long-COVID. *BJGP Open* 2020;**4**:1–12. doi:10.3399/bjgpopen20X101143

39 Mani NS, Budak JZ, Lan KF, *et al.* Prevalence of coronavirus disease 2019 infection and outcomes among symptomatic healthcare workers in Seattle, Washington. *Clin Infect Dis* 2020;**71**:2702–7. doi:10.1093/cid/ciaa761

40 Pimlott N, Agarwal P, McCarthy LM, *et al.* Clinical learnings from a virtual primary care program monitoring mild to moderate COVID-19 patients at home. *Fam Pract* 2020;:1–7. doi:10.1093/fampra/cmaa130

41 Taylor AK, Kingstone T, Briggs TA, *et al.* ‘Reluctant pioneer’: A qualitative study of doctors’ experiences as patients with long COVID. *Heal Expect* 2021;:1–10. doi:10.1111/hex.13223

42 McMichael TM, Currie DW, Clark S, *et al.* Epidemiology of Covid-19 in a Long-Term Care Facility in King County, Washington. *N Engl J Med* 2020;**382**:2005–11. doi:10.1056/nejmoa2005412

43 Fox NS, Melka S. COVID-19 in Pregnant Women: Case Series from One Large New York City Obstetrical Practice. *Am J Perinatol* 2020;**37**:1002–4. doi:10.1055/s-0040-1712529

44 Yan CH, Faraji F, Prajapati DP, *et al.* Self-reported olfactory loss associates with outpatient clinical course in COVID-19. *Int Forum Allergy Rhinol* 2020;**10**:821–31. doi:10.1002/alr.22592

45 Fafi-Kremer S, Bruel T, Madec Y, *et al.* Serologic responses to SARS-CoV-2 infection among hospital staff with mild disease in eastern France. *EBioMedicine* 2020;**59**. doi:10.1016/j.ebiom.2020.102915

46 Andrikopoulou M, Madden N, Wen T, *et al.* Symptoms and critical illness among obstetric patients with coronavirus disease 2019 (COVID-19) infection. *Obstet Gynecol* 2020;**136**:291–9. doi:10.1097/AOG.0000000000003996

47 Brandão Neto D, Fornazieri MA, Dib C, *et al.* Chemosensory Dysfunction in COVID-19: Prevalences, Recovery Rates, and Clinical Associations on a Large Brazilian Sample. *Otolaryngol - Head Neck Surg (United States)* 2021;**164**:512–8. doi:10.1177/0194599820954825

48 Vaes AW, Machado FVC, Meys R, *et al.* Care Dependency in Non-Hospitalized Patients with COVID-19. *J Clin Med* 2020;**9**:2946. doi:10.3390/jcm9092946

49 Barham HP, Taha MA, Hall CA. Does phenotypic expression of bitter taste receptor T2R38 show association with COVID-19 severity? *Int Forum Allergy Rhinol* 2020;**10**:1255–7. doi:10.1002/alr.22692

50 Karimi-Galougahi M, Safavi Naini A, Ghorbani J, *et al.* Emergence and Evolution of Olfactory and Gustatory Symptoms in Patients with COVID-19 in the Outpatient Setting. *Indian J Otolaryngol Head Neck Surg* Published Online First: 2020. doi:10.1007/s12070-020-02166-4

51 Goërtz YMJ, Van Herck M, Delbressine JM, *et al.* Persistent symptoms 3 months after a SARS-CoV-2 infection: the post-COVID-19 syndrome? *ERJ Open Res* 2020;**6**:00542–2020. doi:10.1183/23120541.00542-2020

52 Soffer MD, Shook LL, James K, *et al.* Protocol-Driven Intensive Outpatient Management of Pregnant Patients with Symptomatic Coronavirus Disease 2019. *Open Forum Infect Dis* 2020;**7**:1–7. doi:10.1093/ofid/ofaa524

53 Adhikari EH, Moreno W, Zofkie AC, *et al.* Pregnancy Outcomes among Women with and without Severe Acute Respiratory Syndrome Coronavirus 2 Infection. *JAMA Netw Open* 2020;**3**:1–11. doi:10.1001/jamanetworkopen.2020.29256

54 Ganz-Lord F, Segal KR, Rinke ML. Covid-19 symptoms, duration, and prevalence among healthcare workers in the New York metropolitan area. *Infect Control Hosp Epidemiol* 2020;:1–7. doi:10.1017/ice.2020.1334

55 Petersen MS, Kristiansen MF, Hanusson KD, *et al.* Long COVID in the Faroe Islands: A Longitudinal Study Among Nonhospitalized Patients. *Clin Infect Dis* 2020;:1–18. doi:10.1093/cid/ciaa1792

56 Rubin JE, Crowe SE. COVID-19 Symptoms: Longitudinal Evolution and Persistence in Outpatient Settings. *Ann Intern Med* 2020;**172**:ITC1–14. doi:10.7326/AWED202001070

57 Mania A, Mazur-Melewska K, Lubarski K, *et al.* Wide spectrum of clinical picture of COVID-19 in children — From mild to severe disease. *J Infect Public Health* 2021;**14**:374–9. doi:10.1016/j.jiph.2020.12.029

58 Mendonça CV, Mendes Neto JA, Suzuki FA, *et al.* Olfactory dysfunction in COVID-19: a marker of good prognosis? *Braz J Otorhinolaryngol* 2021;**January**:S1808-8694(20)30240-8. doi:10.1016/j.bjorl.2020.12.002

59 Fisher KA, Olson SM, Tenforde MW, *et al.* Symptoms and recovery among adult outpatients with and without COVID-19 at 11 healthcare facilities—July 2020, United States. *Influenza Other Respi Viruses* 2021;**15**:345–51. doi:10.1111/irv.12832

60 Knight D, Downes K, Munipalli B, *et al.* Symptoms and Clinical Outcomes of Coronavirus Disease 2019 in the Outpatient Setting. *SN Compr Clin Med* 2021;**3**:247–54. doi:10.1007/s42399-021-00746-1

61 Islam MS, Ferdous MZ, Islam US, *et al.* Treatment, persistent symptoms, and depression in people infected with covid-19 in bangladesh. *Int J Environ Res Public Health* 2021;**18**:1–16. doi:10.3390/ijerph18041453

62 Sudre CH, Murray B, Varsavsky T, *et al.* Attributes and predictors of long COVID. *Nat Med* 2021;**27**:626–31. doi:10.1038/s41591-021-01292-y

63 Ronan G, Kumar L, Davey M, *et al.* Factors associated with SARS-CoV-2 infection in patients attending an acute hospital ambulatory assessment unit. *J Med Virol* 2021;**93**:4488–95. doi:10.1002/jmv.26966

64 Mitjà O, Corbacho-Monné M, Ubals M, *et al.* Hydroxychloroquine for Early Treatment of Adults With Mild Coronavirus Disease 2019: A Randomized, Controlled Trial. *Clin Infect Dis* 2020;**0**:1–14. doi:10.1093/cid/ciaa1009

65 Skipper CP, Pastick KA, Engen NW, *et al.* Hydroxychloroquine in Nonhospitalized Adults With Early COVID-19 : A Randomized Trial. *Ann Intern Med* 2020;**173**:623–31. doi:10.7326/M20-4207

66 Tian J, Yan S, Wang H, *et al.* Hanshiyi Formula, a medicine for Sars-CoV2 infection in China, reduced the proportion of mild and moderate COVID-19 patients turning to severe status: A cohort study. *Pharmacol Res* 2020;**161**:105127. doi:10.1016/j.phrs.2020.105127

67 Derwand R, Scholz M, Zelenko V. COVID-19 outpatients: early risk-stratified treatment with zinc plus low-dose hydroxychloroquine and azithromycin: a retrospective case series study. *Int J Antimicrob Agents* 2020;**56**. doi:10.1016/j.ijantimicag.2020.106214

68 Agusti A, Guillen E, Ayora A, *et al.* Efficacy and safety of hydroxychloroquine in healthcare professionals with mild SARS-CoV-2 infection: Prospective, non-randomized trial. *Enferm Infecc Microbiol Clin* Published Online First: 2021. doi:10.1016/j.eimc.2020.10.023

69 Sogut O, Can MM, Guven R, *et al.* Safety and efficacy of hydroxychloroquine in 152 outpatients with confirmed COVID-19: A pilot observational study. *Am J Emerg Med* 2021;**40**:41–6. doi:10.1016/j.ajem.2020.12.014

70 Procter BC, Ross C, Pickard V, *et al.* Clinical outcomes after early ambulatory multidrug therapy for high-risk SARS-CoV-2 (COVID-19) infection. *Rev Cardiovasc Med* 2021;**21**:611–4. doi:10.31083/J.RCM.2020.04.260

71 Rocco PRM, Silva PL, Cruz FF, *et al.* Early use of nitazoxanide in mild Covid-19 disease: randomised, placebo-controlled trial. *Eur Respir J* 2020;:2003725. doi:10.1183/13993003.03725-2020

72 Chen P, Nirula A, Heller B, *et al.* SARS-CoV-2 Neutralizing Antibody LY-CoV555 in Outpatients with Covid-19. *N Engl J Med* 2021;**384**:229–37. doi:10.1056/nejmoa2029849

73 Weinreich DM, Sivapalasingam S, Norton T, *et al.* REGN-COV2, a Neutralizing Antibody Cocktail, in Outpatients with Covid-19. *N Engl J Med* 2021;**384**:238–51. doi:10.1056/nejmoa2035002

74 Cadegiani FA, McCoy J, Gustavo Wambier C, *et al.* Early Antiandrogen Therapy With Dutasteride Reduces Viral Shedding, Inflammatory Responses, and Time-to-Remission in Males With COVID-19: A Randomized, Double-Blind, Placebo-Controlled Interventional Trial (EAT-DUTA AndroCoV Trial – Biochemical). *Cureus* 2021;**13**:1–13. doi:10.7759/cureus.13047

75 Feld JJ, Kandel C, Biondi MJ, *et al.* Peginterferon lambda for the treatment of outpatients with COVID-19: a phase 2, placebo-controlled randomised trial. *Lancet Respir Med* 2021;**9**:498–510. doi:10.1016/S2213-2600(20)30566-X

76 Cadegiani FA, McCoy J, Gustavo Wambier C, *et al.* Proxalutamide Significantly Accelerates Viral Clearance and Reduces Time to Clinical Remission in Patients with Mild to Moderate COVID-19: Results from a Randomized, Double-Blinded, Placebo-Controlled Trial. *Cureus* 2021;**2**:1–8. doi:10.7759/cureus.13492

77 López-Medina E, López P, Hurtado IC, *et al.* Effect of Ivermectin on Time to Resolution of Symptoms among Adults with Mild COVID-19: A Randomized Clinical Trial. *JAMA - J Am Med Assoc* 2021;**325**:1426–35. doi:10.1001/jama.2021.3071

78 Roostaei Firozabad A, Meybodi ZA, Mousavinasab SR, *et al.* Efficacy and safety of Levamisole treatment in clinical presentations of non-hospitalized patients with COVID-19: a double-blind, randomized, controlled trial. *BMC Infect Dis* 2021;**21**:1–8. doi:10.1186/s12879-021-05983-2

79 Jagannathan P, Andrews JR, Bonilla H, *et al.* Peginterferon Lambda-1a for treatment of outpatients with uncomplicated COVID-19: a randomized placebo-controlled trial. *Nat Commun* 2021;**12**:1–10. doi:10.1038/s41467-021-22177-1
