# Supplementary material for: Management of symptomatic patients with suspected mild-moderate COVID-19 in general practice. What was published within the first year of the pandemic? A scoping review
Source: Eur J Gen Pract. 2021 Nov 18;27(1):339–45. doi: 10.1080/13814788.2021.2002295 (PMC8604528; doi:10.1080/13814788.2021.2002295)
Supplement: Supplemental Appendix 1: search string [file IGEN_A_2002295_SM1621.docx]

# Appendix A

Search string pubmed

(((wuhan[All Fields] AND ("coronavirus"[MeSH Terms] OR "coronavirus"[All Fields])) AND 2019/12[PDAT] : 2030[PDAT]) OR 2019-nCoV[All Fields] OR 2019nCoV[All Fields] OR COVID-19[All Fields] OR SARS-CoV-2[All Fields])

AND

(“mild”[TiAb] OR “moderate”[TiAb])

AND

(“family”[all fields] OR physician*[all fields] OR practice*[tw] OR “primary care”[all fields] OR “Primary Health Care”[mh] OR primary[tw] OR general pract*[tiab] OR gp[tiab] OR gps[tiab] OR “non-hospitalized”[TiAb] OR “non-hospitalised”[TiAb] OR “outpatient*”[TiAb] OR “ambulatory”[TiAb])

NOT "Review"[pt] NOT “systematic review”[Pt] NOT "Support of Research"[Pt] NOT "Case Reports"[Pt]
